# Feasibility and potential value of a local governmental frail check-up program for the risk assessment of long-term care in apparently healthy older citizens: a prospective study

**DOI:** 10.1186/s12913-025-12918-z

**Published:** 2025-05-22

**Authors:** Yoji Nagai, Yasumasa Kakei, Tatsuo Kagimura, Shinsuke Kojima, Hisatomo Kowa, Tohmi Osaki, Ryoma Kayano, Yasuji Yamamoto

**Affiliations:** 1 Department of Clinical Research Facilitation, Institute for Advancement of Clinical and Translational Science, Kyoto University Hospital, 54 Kawahara-cho, Shogoin, Sakyo-ku, Kyoto, 606-8507 Japan; 2 Division of Translational Science, Kobe University Graduate School of Medicine, 7-5-2 Kusunoki-cho, Chuo-ku, Kobe, 650-0017 Japan; 3 Clinical & Translational Research Center, Kobe University Hospital, 7-5-2 Kusunoki-cho, Chuo- ku, Kobe, 650-0017 Japan; 4 Translational Research Center for Medical Innovation, Foundation for Biomedical Research and Innovation, 1-5-4 Minatojima-minamimachi, Chuo-ku, Kobe, 650-0047 Japan; 5 Division of Cognitive and Psychiatric Rehabilitation, Department of Rehabilitation Science, Kobe University Graduate School of Health Sciences, 7-10-2 Tomogaoka-cho, Suma-ku, Kobe, 654-0142 Japan; 6 Faculty of Rehabilitation, Kobe Gakuin University, 518 Arise, Ikawadani-cho, Nishi-ku, Kobe, 651-2180 Japan; 7 Department of Psychiatry, Kobe University Graduate School of Medicine, 7-5-2 Kusunoki-cho, Chuo-ku, Kobe, 650-0017 Japan; 8 World Health Organization Centre for Health Development (WHO Kobe Centre), I.H.D. Center Building 9 F, 1-5-1 Wakinohama-Kaigandori, Chuo-ku, Kobe, 651-0073 Japan; 9 Department of Biosignal Pathophysiology, Kobe University Graduate School of Medicine, 7-5- 2 Kusunoki-cho, Chuo-ku, Kobe, 650-0017 Japan; 10 Medical Center for Student Health, Kobe University, 1-1 Rokodai-cho, Nada-ku, Kobe, 657- 8501 Japan

**Keywords:** Long-term care, General frailty, Health check-up, Local governmental services, Aging

## Abstract

**Background:**

Earlier identification of individuals at risk of needing long-term care can increase the opportunities for preventive/therapeutic interventions, leading to a reduced social burden.

**Methods:**

We examined the feasibility and potential value of a frailty check-up program implemented by the local government for risk assessment of long-term care needs in 1,528 apparently healthy older (aged 64/65 years) citizens in Kobe, Japan, between August 2017 and March 2018. The program comprised a questionnaire on general frailty (Comprehensive score) and evaluation of other frailty-related measures, cognitive function-related activities of daily living (Cognitive Function Instrument), and health-related quality of life (EQ-5D-5L). In March 2020, these measures were merged with data on long-term care or support certification, where the latter represented a condition requiring care for a certain period preceding long-term care needs.

**Results:**

Overall, 15 citizens were certified as having long-term care needs and 38 as having long-term care or support needs. Using receiver operating characteristic curve analysis, certain measures, including the Comprehensive score and EQ-5D-5L, significantly predicted the incidence of long-term care or support needs.

**Conclusions:**

The frailty checkup program conducted by the local government may be feasible and valuable for risk assessment of long-term care needs in apparently healthy older citizens.

**Supplementary Information:**

The online version contains supplementary material available at 10.1186/s12913-025-12918-z.

## Background

The World Health Organization defines LTC as a broad range of personal, social, and medical services and support that ensure people with, or at risk of, a significant loss of intrinsic capacity (due to mental or physical illness and disability) can maintain a level of functional ability consistent with their basic rights and human dignity” [1]. Across the Organisation for Economic Co-operation and Development countries, approximately 10.7% of people aged ≥ 65 years receive long-term care (LTC) in either home or LTC facilities [2]. In recent decades, the Japanese population has experienced an increasing need for LTC [3], requiring efficient adaptation by healthcare systems. In association with such a situation, the LTC Insurance Act was implemented in Japan in 1997 [4]; thereafter, the public certification of LTC needs has been implemented in combination with the certification of support needs, where the latter is defined as a health status transiently requiring care for a certain period preceding the LTC needs.

Various underlying conditions including general frailty and cognitive impairment predispose individuals to LTC or support needs. Although frailty is defined in various ways, a commonly accepted definition is a state of increased vulnerability to poor resolution of homeostasis after a stressor event, which increases the risk of adverse outcomes, including falls, delirium, and disability [5]. Asymptomatic frailty in healthy older individuals is associated with future LTC needs. Since the earlier identification of such conditions may increase the chance of preventive and therapeutic interventions, potentially leading to the reduction of LTC needs, Kobe City started a frailty check-up program known as “Frail Kenshin” in 2017. To improve the health of older citizens, the program included a simple questionnaire on general frailty, as defined by the Comprehensive score, and evaluation of other frailty-related measures such as motor function, dental health, oral function, swallowing ability, chewing ability, and grip strength. Of note, Comprehensive score, a modification of “Kihon Checklist” [6], was a measure devised by the Kobe city, to be used for the “Frail Kenshin” program.

Simultaneously, recent studies have demonstrated a close link between general frailty, decrease in activities of daily living (ADL), and cognitive impairment [7, 8]. Furthermore, dementia is a major cause of LTC needs, emphasizing the importance of adapting measures to health systems [9]. In the paucity of effective disease-modifying approaches for dementia [10], increasing evidence suggests the positive effects of non-pharmacological interventions, such as exercise, cognitive training, and maintenance of ADL [4, 11, 12]. Moreover, subjective health-related quality of life (QOL) can serve as an indicator of the risk of LTC or support needs. As an indicator of earlier ADL impairments due to cognitive impairment, the Cognitive Function Instrument (CFI) is a simple self-administered questionnaire that can be utilized without an interview [13, 14]. Additionally, as an internationally used indicator of health-related QOL, the EQ-5D-5L consists of simple questions on mobility, self-care, usual activities, pain/discomfort, and anxiety/depression, yielding a single index value that reflects the satisfaction level with an individual’s current health condition [15].

Using frailty-related questionnaires and measures, this study examined the feasibility and potential value of a locally conducted frailty checkup program for apparently healthy older citizens as a risk assessment of future LTC needs.

## Methods

### Subjects

This study is a component of the “Kobe Project for the Exploration of Newer Strategies to Reduce the Social Burden of Dementia,” a collaborative research study by three local institutions and the World Health Organization that received strong support from Kobe City. The core concepts and outline of this research project have been reported previously [16]. Under the framework of this research project, the current study evaluated the 2-year incidence of LTC or support needs in community-dwelling citizens who participated in the frailty checkup program provided by Kobe City.

In Kobe City, approximately 20,000 citizens turn 65 years of age annually. The frailty check-up program was offered to citizens aged 64 or 65 years (approximately 6, 000 individuals) who were covered by the “Kokumin Kenko Hoken” (a Japanese national health insurance system). To explore the potential utility of the frailty checkup program for the risk assessment of LTC/support needs, the current prospective study was conducted on 1,528 citizens (aged 64/65 years; male: 36%, female: 64%) who voluntarily participated in the program between August 2017 and March 2018.

### Evaluated measures

#### Comprehensive score and other measures on general frailty

The frailty check-up program is a health check-up program for older citizens that started in Kobe City in 2017 and is held at various venues in the respective districts on several occasions [16]. In the program, individuals are evaluated based on a frailty check sheet, which includes self-assessed questionnaires and physically assessed measures of general frailty. Specifically, self-assessed frailty was evaluated by the Comprehensive score, which is a modification of the “Kihon Checklist” [6] and comprises 20 simple questions on general frailty (Additional file 1), with the score calculated by the number of “yes” answers. Physical measures included motor function, oral function, chewing ability, dental health, swallowing ability, grip strength, and body mass index (Additional file 2). During the program, a frailty check sheet was distributed to the participants and each participant was asked to answer a questionnaire in writing onto the sheet. Thereafter, the filled sheets were retrieved and visually checked by the person in charge, where blank items were orally re-asked and compensated as possible. Subsequently, measures of physical frailty were evaluated in the same venue.

#### Cognitive function instrument (CFI)

The CFI is a screening tool developed by the US Alzheimer’s Disease Cooperative Study group over 10 years [13]. It is a simple questionnaire consisting of “Self” and “Partner” reports, which can be administered by mail, over the phone, or online, requiring no in-person interview. It comprises 14 questions related to changes in functional ability due to cognitive decline. CFI scores are calculated by summing 1 point for “Yes,” 0 for “No,” and 0.5 for “Maybe.” The CFI questionnaire includes “Self” and “Partner” versions, but the current study utilized only the “Self” version because the study setting did not allow for the use of the “Partner” version. CFI questionnaire was carried out at the venue of Frail Kenshin program, and the filled questionnaire was retrieved by the person in charge. In addition, Japanese translations were used in this study.

#### EQ-5D-5L

The EQ-5D is a standardized measure of health status developed by the EuroQol Group to provide a simple generic measure of health for clinical and economic appraisals. It is designed for self-completion, is used worldwide as an indicator of health-related QOL, and is delivered in many settings, including postal surveys, clinical settings, and in-person interviews [15]. Notably, the EQ-5D has been published in 171 languages; the official Japanese version was used in this study. Although the EuroQol Group provides two versions of the EQ-5D with three or five dimensions, the latter (EQ-5D-5L) was used in this study. The EQ-5D-5L includes five categories with five questions each, for a total of 25 questions. Based on the pattern of answers, the health status of an individual is given as a single index value ranging from 0 to 1 (death = 0; complete health = 1). Additionally, based on how good or bad an individual feels, his/her current health status is rated using a visual analog scale ranging from 0 to 100 (worst health he/she can imagine = 0, best health he/she can imagine = 100). EQ-5D-5L questionnaire was carried out at the venue of Frail Kenshin program, and the filled questionnaire was retrieved by the person in charge.

### Certification of LTC or support needs

The certification of LTC/support needs has been implemented in Japan via the National LTC Insurance Act since April 2000 [4]. The principal beneficiaries were individuals aged ≥ 65 years. Applications are submitted by citizens who wish to be certified for them to receive the benefits of utilizing healthcare services at a much lower rate. Upon receiving applications from citizens, the health and living conditions of the applicants were comprehensively evaluated by the municipal office according to the Ministry Ordinance of the Japanese Government [17], and certifications were performed based on predetermined algorithms. Final judgments were made by a publicly authorized committee of the city. Thus, the criteria for LTC need certification was based on administrative purposes of the Japanese government, but not on established scales or specific parameters on ADL.

Following the certification of LTC needs, there are five levels of categorization depending on the length of time requiring daily care; the levels range from “LTC level 1” (least severe) to “LTC level 5” (most severe; Additional file 3). Similarly, following the definition of “Needed Support Condition” in the LTC Insurance Act, support needs are certified when an individual is in the condition assumed to require care during a certain period to reduce or prevent aggravation of the condition, or in the condition assumed to cause difficulties in performing daily activities during a certain period (Additional file 3).

As part of the certification process, levels of impairment in ADL associated with dementia are also evaluated, with possible categories ranging from “None” to “Grade IV” (Additional file 4). With the use of this categorization, “LTC need with dementia” was defined in this study as LTC needs alongside dementia-related ADL impairments at “Grade IIa” or worse. This is in accordance with our previous report [18], where “Grade IIa” represents a condition wherein the individual remains independent even though precautions are required outside the home, and despite the presence of symptoms or behaviors interfering with daily life or making communications difficult.

### Data management

Data on Comprehensive scores and other frailty-related measures, CFI, and EQ-5D-5L were collected during the frailty check-up program, and the information was recorded on the frailty check sheet. These data were electronically recorded and retained in a database dedicated to municipal offices.

Certification of LTC/support needs under the LTC Insurance Act was routinely conducted at the municipal office, and the above data were merged with the LTC/support needs certification data at the end of March 2020. Subsequently, the merged dataset was transferred from the municipal office to our study group after removing all personal information.

### Ethical considerations

This study was approved by the WHO ethics committee (ERC.0002899) and the Ethics Review Committee of Kobe University (approval no. 170018). This study conformed to the provisions of the Declaration of Helsinki and was conducted in accordance with Japan’s “Ethical Guidelines for Medical and Health Research Involving Human Subjects.” As this was an observational study, the Kobe University Ethics Committee waived the requirement for obtaining informed consent from the study participants. All information on each individual was collected in compliance with the law on the protection of personal information (May 30, 2003, Law No. 57) after providing a simple explanation of the purpose.

### Statistical analysis

The cumulative incidence of LTC needs or LTC/support needs certification was calculated by the Kaplan–Meier method. A univariate logistic analysis, using LTC needs or LTC/support needs certification as the objective variable and frailty check items and others as explanatory variables, was carried out to generate a receiver operating characteristic (ROC) curve, and thereafter the area under the curve (AUC) and its 95% confidence interval were calculated, where AUC = 0.5 was tested as the null hypothesis. The variables that created the ROC curve were Comprehensive score, 25-item food intake questionnaire score, Number of remaining teeth, Calf circumference, Finger-circle test score, Color-changeable Chewing gum test score, Repetitive saliva swallowing test, Hand grip strength, Chair standing test score, CFI score, EQ-5D-5L index value, EQ-5D-5L VAS, Height, Body weight and BMI. Also, a multivariate logistic analysis was performed with all these variables as covariates, and thereafter ROC curves were obtained from the full model estimates. Additionally, variables were selected using a stepwise method, and ROC curves were obtained using estimates of the selected variable models. All analyses were conducted using SAS version 9.4 (Cary, NC, USA). The level of significance was set at *p* < 0.05 (2-tailed).

## Results

### Incidence of long-term care or support needs certification

**Fig. 1:**
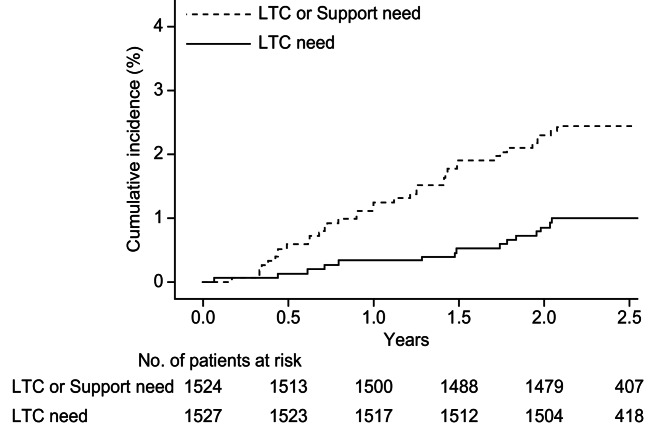
Cumulative incidence of long-term care or support need certification. LTC: Long-term care: Among the enrolled 1,528 citizens, one citizen was found to be in LTC need and three citizens were in support need at the time of enrolment and were excluded from the analyses

The cumulative incidence of LTC needs certification gradually increased over time, as did the combined cumulative incidence of LTC/support needs (Fig. 1). During the follow-up period, 15 individuals were certified to be in LTC needs (Table 1), with an actual incidence of 0.42 (95% CI:0.23 to 0.69) per 100 person-years. Also, 38 individuals were certified to be in LTC/support needs, with an actual incidence of 1.08 (95% CI:0.76 to 1.48) per 100 person-years. Additionally, among the 15 individuals in whom LTC needs was certified, 8 individuals were found to be in “LTC need with Dementia.”

**Table 1:** Cumulative incidence of long-term care/support needs certification

	LTC needs	LTC or Support needs*
Number of individuals	15 (men: 4, women:11)	38 (men: 11, women:27)
Category		
Support needs	-	29 (76.3%)
LTC needs level 1	8 (53.3%)	5 (13.2%)
LTC needs level 2	5 (33.3%)	2 (5.3%)
LTC needs level 3	1 (6.7%)	1 (2.6%)
LTC needs level 4	1 (6.7%)	1 (2.6%)

Explanations of these categories are provided in Additional file 3

*LTC* long-term care

*The first certification of either LTC or Support needs was counted, and values in the respective categories represent the number of such certifications during the follow-up period. (Note that among the 29 individuals initially certified to be in support needs, three individuals subsequently shifted to LTC needs level 1 and three individuals shifted to LTC needs level 2)

### Prediction of long-term care or long-term care/support needs

Using ROC curve analysis derived from univariate logistic regression, the AUC of certain measures was significantly larger than 0.5 for the prediction of LTC needs (Table 2). The largest AUC was found for the Comprehensive score, followed by the Finger-circle test score, EQ-5D-5L index value, and Calf circumference. In addition, the AUC of certain measures was significantly larger than 0.5 for the prediction of LTC/support needs (Table 3). The largest AUC was found for the EQ-5D-5L index value, followed by the Comprehensive, EQ-5D-5L VAS, and CFI scores. Additionally, although the CFI score significantly predicted the occurrence of LTC/support needs, its predictive value was limited.

**Table 2:** Areas under the receiver operating characteristics curve for the incidence of long-term care need

		AUC of ROC
Univariate analysis	Frail check items	
	Comprehensive score	0.843*
	25-item food intake questionnaire score	0.492
	Number of remaining teeth	0.620
	Calf circumference (cm)	0.777*
	Finger-circle test score	0.780*
	Color-changeable Chewing gum test score	0.511
	Repetitive saliva swallowing test	0.554
	Hand grip strength (kg)	0.582
	Chair standing test score	0.711
	CFI score	0.668
	EQ-5D-5L utility index	0.780*
	EQ-5D-5L VAS	0.648
	Height (cm)	0.617
	Weight (kg)	0.617
	BMI	0.635
Multivariate analysis (Full model)	All above parameters	0.938*
Multivariate analysis (Stepwise model**)	Comprehensive score, Finger-circle test score	0.881*

As artificially created rating scales, the Cronbach's alpha of the Comprehensive score, the 25-item food intake questionnaire score and the CFI score in this study were 0.610, 0.894 and 0.812, respectively

*AUC* area under the curve, *ROC* receiver operating characteristics, *BMI* body mass index, *CFI* cognitive function instrument, *VAS* visual analogue scale

*P<0.05, **Shown items are the combination that achieved the largest AUC after stepwise elimination. Explanations of the respective categories are provided in Additional files 1 and 2

**Table 3:** Areas under the receiver operating characteristics curves for the combined incidence of long-term care or support need

		AUC of ROC
Univariate analysis	Frail check items	
	Comprehensive score	0.741*
	25-item food intake questionnaire score	0.482
	Number of remaining teeth	0.578
	Calf circumference (cm)	0.600
	Finger-circle test	0.651
	Color-changeable Chewing gum test sore	0.530
	Repetitive saliva swallowing test	0.533
	Hand grip strength (kg)	0.605
	Chair standing test score	0.483
	CFI score	0.623*
	EQ-5D-5L utility index	0.834*
	EQ-5D-5L VAS	0.688*
	Height (cm)	0.640
	Weight (kg)	0.521
	BMI	0.529
Multivariate analysis (Full model)	All above parameters	0.858*
Multivariate analysis (Stepwise model**)	Calf test score, Finger-circle test score, BMI, EQ-5D-5L utility value, female sex	0.862*

As artificially created rating scales, the Cronbach's alpha of the Comprehensive score, the 25-item food intake questionnaire score and the CFI score in this study were 0.610, 0.894 and 0.812, respectively

*AUC* area under the curve, ROC: receiver operating characteristics, *BMI* body mass index, *CFI* cognitive function instrument, *VAS* visual analogue scale

*P<0.05, ** Shown items are the combination that achieved the largest AUC after stepwise elimination. Explanations of the respective categories are provided in Additional files 1 and 2

To further evaluate the predictive value of respective parameters for LTC needs or LTC/support needs, multivariate analysis was performed, although it was preliminary because of the limited number of events and possible sample bias. For the prediction of LTC needs, AUC of the full model including all parameters for LTC needs was 0.938, and in the selected variable model the combination of the Comprehensive score and Finger-circle test score demonstrated the largest AUC (0.881) (Table 2). Also, AUC of the full model for LTC/support needs was 0.858, and in the selected variable model the combination of Calf circumference, Finger-circle test score, BMI, EQ-5D-5L index value and Female sex demonstrated the largest AUC (0.862) (Table 3).

## Discussion

In this study, we demonstrated the feasibility and potential value of a locally conducted frailty checkup program in contributing to LTC/support needs risk assessments. We are unaware of previous studies that have linked frailty check-up data with future LTC needs by utilizing existing activities taking place within the local government.

Due to the administrative setting of Kobe City, the subjects were relatively young (64 or 65 years old) for a study linking current frailty and near-future LTC/support needs. Even under such conditions, a certain proportion of individuals shifted to the LTC/support needs condition, and the cumulative incidence gradually increased during the study period (Fig. 1). This finding suggests a potential need for administrative interventions, even at an earlier stage of aging, to reduce the social burden of LTC/support. Additionally, approximately 50% (8 of 15) of individuals who shifted to the LTC needs condition had ADL impairment due to dementia, suggesting an interactive association between general frailty and dementia [7, 8, 19], although the presence of a causal relationship must be examined.

In univariate ROC curve analysis, certain measures were predictive of LTC needs (Table 2) and also of LTC/support needs (Table 3). Among the measures considered, the comprehensive score and EQ-5D-5L index had the highest predictive value for LTC and LTC/support needs, respectively. This finding corresponds with those of other studies from Japan [20–23] indicating an association between subjective frailty and near-future LTC needs. In addition to LTC needs alone, we analyzed the combined incidence of LTC/support needs because support needs represent a condition nearly equivalent to LTC needs that could only be temporary or reversible by social or medical interventions, preceding the development of a persistent LTC needs. However, these findings are preliminary, requiring additional investigation to clarify their implications for LTC/support risk assessment.

In addition, ROC curve analysis derived from multivariate logistic regression revealed that the combination of the Comprehensive score and finger-circle test scores was the most predictive of the need for LTC. Of note, AUC value obtained by univariate analysis using the Comprehensive score was sufficiently large, which was not surprising because it is an integrated measure that reflects various aspects of frailty. Furthermore, although the highest predictive values for LTC/support needs were found for the combination of several measures in the frailty checkup, the associated AUC was only slightly larger than that found for the EQ-5D-5L alone. Based on these findings, a suboptimal risk assessment could be achieved by selecting a simple subjective measure rather than a combination of multiple objective measures, although meticulous investigations are still required. Additionally, the predictive value of the CFI score, which is an indicator of ADL impairment due to dementia, was not significant for LTC needs and was limited to LTC/support needs. Further investigations targeting a population one decade older may be required to adequately evaluate the impact of dementia on future LTC/support needs.

The current study has some limitations. First, the number of participants with identified LTC/support needs was relatively small, and the duration of follow-up was short (2 years), limiting the statistical power of this study. Second, the targeted population was relatively young because of the utilization of existing administrative practices, which precludes efficient assessments of the link between frailty/dementia and LTC/support needs. Accordingly, the results of this study should be interpreted as preliminary and require further studies targeting older and larger populations with longer follow-up periods. Third, the Comprehensive score used in this study is a measure newly devised by the Kobe City to be used for the current health checkup program, in which blank answers were orally asked and compensated as possible during the checkup, requiring careful consideration when used by other municipalities and limiting its unconditional generalizability. Despite these limitations, this study demonstrates an example of an operational model of frailty check-up programs for future LTC/support needs risk assessment in apparently healthy older citizens by utilizing the existing civil services of the local government.

## Conclusions

The frailty checkup program performed by the local government may be feasible and facilitate risk assessments of future LTC needs among apparently healthy community-dwelling older citizens, which could serve as an administrative operational model toward the final goal of reducing the social burden of LTC.

## Supplementary Information

Additional file 1. Comprehensive score. Full list of items of the Comprehensive score.

Additional file 2. Explanation of frailty check items. An explanation of the different components of the frailty check.

Additional file 3. Types of long-term care or support need. An overview of exemplar indicative conditions related to each category of long-term care or support needs.

Additional file 4. Daily life independence level with dementia. Description of the different grades comprising the daily life independence level with dementia assessment.

## Data Availability

The datasets generated and/or analyzed during the current study are not publicly available because of a written agreement between Kobe University and the Foundation for Biomedical Research and Innovation; however, the datasets are available from the corresponding author upon reasonable request.

## Abbreviations

AUC: Area under the Curve
BMI: Body Mass Index
CFI: Cognitive Function Instrument
EQ-5D-5L: EuroQol Five Dimensions
LTC: Long-term Care
QOL: Quality of Life
ROC: Receiver Operating Characteristic
