# Supplementary material for: Feasibility and potential value of a local governmental frail check-up program for the risk assessment of long-term care in apparently healthy older citizens: a prospective study
Source: BMC Health Serv Res. 2025 May 22;25:743. doi: 10.1186/s12913-025-12918-z (PMC12096563; doi:10.1186/s12913-025-12918-z)
Supplement: Supplementary file 1 — Additional file 1. Comprehensive score. Full list of items of the Comprehensive score. [file 12913_2025_12918_MOESM1_ESM.docx]

**Additional File 1. Comprehensive score**

| Q1 | Do you take the bus or train by yourself? |
| --- | --- |
| Q2 | Do you go shopping to buy daily necessities by yourself? |
| Q3 | Do you manage your own deposit and savings at the bank? |
| Q4 | Do you sometimes visit your friends? |
| Q5 | Do you turn to your family or friends for advice? |
| Q6 | Do you go out at least once a week? |
| Q7 | Do you go out less frequently compared to last year? |
| Q8 | Do your family or your friends point out your memory loss? E.g. “You ask the same question over and over again”. |
| Q9 | Do you make a call by looking up phone numbers? |
| Q10 | Do you find yourself not knowing today’s date? |
| Q11 | Do you normally climb stairs without using handrails or wall for support? |
| Q12 | Do you normally stand up from a chair without any aids? |
| Q13 | Do you normally walk continuously for 15 minutes? |
| Q14 | Have you experienced a fall in the past year? |
| Q15 | Do you have a fear of falling while walking? |
| Q16 | Have you lost 2 kg or more in the past 6 months? |
| Q17 | Do you have any difficulties eating tough foods compared to 6 months ago? |
| Q18 | Have you choked on your tea or soup recently? |
| Q19 | Do you often experience having a dry mouth? |
| Q20 | Height: cm, weight: kg, BMI: kg/m^2^ If BMI is less than 18.5, this item is scored |

Comprehensive score is calculated to be the number of “yes” answers, which was proposed by the Kobe city municipal office, as a modification of the “Kihon Checklist” (Arai H, Satake S. English translation of the Kihon Checklist. Geriatr Gerontol Int. 2015;15:518–9).
