# Supplementary material for: Feasibility and potential value of a local governmental frail check-up program for the risk assessment of long-term care in apparently healthy older citizens: a prospective study
Source: BMC Health Serv Res. 2025 May 22;25:743. doi: 10.1186/s12913-025-12918-z (PMC12096563; doi:10.1186/s12913-025-12918-z)
Supplement: Supplementary file 2 — Additional file 2. Explanation of frailty check items. An explanation of the different components of the frailty check. [file 12913_2025_12918_MOESM2_ESM.docx]

**Additional File 2. Explanation of frailty check items**

| Frailty check items | Explanation |
| --- | --- |
| 25-item food intake  questionnaire score | In accordance with Koshino^1)^, 25 kinds of food were selected, to each of which 2 points were assigned when “easy to eat”, 1 point when “difficult to eat” and 0 point when “unable to eat”. The score was calculated to be the sum of respective points for each 25-item food, ranging from 0 to 50 points for an individual. |
| Finger-circle test score | In accordance with Hiraoka^2)^, this test enables a simple assessment of sarcopenia, by checking whether the thickest part of the calf is smaller or larger than, or just-fits to the ring made by the thumb and forefinger of both hands. The test comprises 3 levels: 1 point when the ring cannot be closed (i.e., The calf circumference is larger than the ring), 2 points when the ring fits the calf circumference, and 3 points when an interstice exists between the ring and calf circumference. |
| Color-changeable  Chewing gum test score | In accordance with Kamiyama^3)^, the strength of chewing was estimated by the color of chewed gum and expressed by 5 levels: 5 points were assigned for “green” (weakest chewing ability), 4 points for “yellow”, 3 points for “light cherry”, 2 points for “dark cherry”, and 1 point for “red” (strongest chewing ability). |
| Repetitive saliva  swallowing test score | In accordance with Horiguchi^4)^, the swallowing function was assessed by the number of times saliva was swallowed in 30 seconds; a score of 1 was assigned for 2 or more times, and 0 for 0 or 1 time. |
| Chair standing test score | In accordance with the method by Macfarlane^5)^, ability of standing up was evaluated by the number of times an individual could stand up from a typical height chair within 15 seconds: 1 point was assigned for 12 or more times, 2 points for 9 to 11 times, 3 points for 8 times, 4 points for 5–7 times, and 5 points for 4 times or less. |
| **1)** Koshino H, Hirai T, Yokoyama Y, Tanaka M, Toyoshita Y, Iwasaki K, Sudo E. Mandibular residual ridge shape and the masticatory ability in complete denture wearers. Nihon Hotetsu Shika Gakkai Zasshi. 2008;52:488–93.  **2)** Hiraoka A, et al. Easy surveillance of muscle volume decline in chronic liver disease patients using finger-circle (yubi-wakka) test. J Cachexia Sarcopenia Muscle. 2019;10:347–54.  **3)** Kamiyama, M. Kanazawa M, Fujinami Y, Minakuchi S. Validity and reliability of a Self-Implementable method to evaluate masticatory performance: Use of color-changeable chewing gum and a color scale. J Prosthodont Res. 2010;54:24–8.  **4)** Horiguchi S, Suzuki Y. Screening tests in Evaluating Swallowing Function. Japan Med Assoc J. 2011;1:31–4.  **5)** Macfarlane DJ, Chou KL, Cheng YH, Chi I. Validity and normative data for thirty-second chair stand test in elderly community-dwelling Hong Kong Chinese. Am J Hum Biol. 2006;18:418–21. | |
