# Supplementary material for: Feasibility and potential value of a local governmental frail check-up program for the risk assessment of long-term care in apparently healthy older citizens: a prospective study
Source: BMC Health Serv Res. 2025 May 22;25:743. doi: 10.1186/s12913-025-12918-z (PMC12096563; doi:10.1186/s12913-025-12918-z)
Supplement: Supplementary file 3 — Additional file 3. Types of long-term care or support need. An overview of exemplar indicative conditions related to each category of long-term care or support needs. [file 12913_2025_12918_MOESM3_ESM.docx]

**Additional File 3. Types of long-term care or support need**

| Category | Indicative conditions (example) |
| --- | --- |
| Support need level 1 | The person can rise, walk, and perform most other essential daily life activities by himself/herself. However, the person needs some support for task-based activities in daily life, including cooking, shopping and taking oral medicine. |
| Support need level 2 | The person’s ability to handle task-based activities in daily life is slightly lower than that of individuals in the Support need level 1 category, and he/she needs more support. |
| LTC need level 1 | The person faces difficulty in performing essential daily life activities by himself/herself. The person’s ability to handle task-based activities in daily life is lower than that of individuals in the Support need category. |
| LTC need level 2 | The person is in a state similar to that detailed under LTC need level 1, but requires more care to be able to perform essential daily life activities. |
| LTC need level 3 | Compared with LTC need level 2, the person’s abilities to perform essential daily life activities and task based activities are significantly lower. As a result, he/she requires almost constant care. |
| LTC need level 4 | The person is in a state similar to that detailed under LTC need level 3, but his/her ability to act is lower. As a result, he/she faces difficulty living without constant care. |
| LTC need level 5 | The person’s ability to act is even lower than that of individuals in the LTC need level 4 category. As a result, he/she requires almost constant care to live. |

LTC: long-term care, Modified from the “Guidebook for Long-term Care Insurance. Shinjuku City Long-term Care Insurance Division, Fiscal 2018” (http://www.foreign.city.shinjuku.lg.jp/en/wp-content/uploads/sites/4/2018/10/kaigo2018_e.pdf)
