# Supplementary material for: Feasibility and potential value of a local governmental frail check-up program for the risk assessment of long-term care in apparently healthy older citizens: a prospective study
Source: BMC Health Serv Res. 2025 May 22;25:743. doi: 10.1186/s12913-025-12918-z (PMC12096563; doi:10.1186/s12913-025-12918-z)
Supplement: Supplementary file 4 — Additional file 4. Daily life independence level with dementia. Description of the different grades comprising the daily life independence level with dementia assessment. [file 12913_2025_12918_MOESM4_ESM.docx]

**Additional File 4. Daily life independence level with dementia**

| Grade I | Have some sort of dementia but is almost independent in daily life of domestic and social activities. |
| --- | --- |
| Grade II | Symptoms, behavior or difficulty in communication that interfere with the person’s daily life are observed in some degree but can live independently if looked after by someone.  IIa: With the condition described above outside the home.  IIb: With the condition described above even at home. |
| Grade III | Symptoms, behavior or difficulty in communication that interfere with the person’s daily life are observed occasionally, and requires care.  IIIa: With the condition described above mainly during the daytime.  IIIb: With the condition described above mainly at night. |
| Grade IV | Symptoms, behavior or difficulty in communication that interfere with the person’s daily life are observed frequently, and requires constant care. |

(https://www.mhlw.go.jp/english/database/db-hss/dl/siel-2010-04.pdf)
